# Dynamic assessment of eco-civilization in Guangdong Province based on geographic information system (GIS) and analytic hierarchy process (AHP)

**DOI:** 10.1016/j.heliyon.2023.e22579

**Published:** 2023-11-21

**Authors:** Qing Guo, Mingshan Liu

**Affiliations:** School of Economics and Trade, Guangdong University of Foreign Studies, Guangzhou, 510006, China

**Keywords:** Guangdong province, Ecological civilization, Dynamic evaluation, GIS, AHP

## Abstract

Based on the data from 2017 to 2021, this paper uses the organic combination of Geographic Information System (GIS) and Analytic Hierarchy Process (AHP) to conduct a dynamic evaluation of the standard of Guangdong's ecological civilization. The results clearly reveal that: (1) the Guangdong Province's ecological civilization is 46.31 % above the Chinese average, but growth is uneven across cities; (2) the fluctuation of the level of eco-civilization building in the province declines more obviously; (3) cities perform differently in different dimensions of eco-civilization building, most cities in Guangdong exhibited positive performance in terms of ecology and environment, but poor performance in terms of economic development and social construction; (4) economic benefits have a strong positive influence on the level of eco-civilization building in Guangdong Province. Finally, targeted suggestions are made for the construction of eco-civilization in Guangdong Province.

## 1 Introduction

Resources and the environment have laid the foundation for the birth and development of human beings' civilization (Summers et al., 2012) [1]. Griggs et al. (2013) [2] also emphasises the importance of a healthy ecology for the sustainable advancement of society. Accelerating the development of ecological civilization has become a consensus in the world. The report of the Twentieth National Congress of the Communist Party of China emphasises that nature is the basic condition on which human beings rely for survival and development, and that respecting nature, adapting to nature, and protecting nature are the inherent requirements for building a modern socialist country in an all-round way, which shows the importance of ecological civilization for building a modernisation in which human beings and nature coexist in a harmonious way. Foreign countries in the study of ecological civilization, on the other hand, believe that ecological civilization is the ultimate goal of a society and the unity of socialism and ecological principles (Zhou & Jiang, 2023) [3]. In a broad sense, ecological civilization includes the synthesis of economic, educational, political, agricultural and other social reforms (Philip et al., 2014) [4]. Guangdong Province is one of the most economically developed provinces in China. With the rapid development of society and economy, environmental problems are emerging, such as: air pollution, water pollution, solid waste pollution, etc. At the same time, Guangdong Province, as a pioneering area of China's reform and opening up, is also facing the strategic task of building an “eco-civilization” in the process of achieving sustainable economic development (Wu et al., 2017) [5]. Although the Guangdong government has actively introduced the “14th Five-Year Plan for the Establishment of Eco-Civilization in Guangdong”, aiming to basically build an ecological civilization system, reasonably allocate energy resources, and build a beautiful Guangdong, it is a new challenge to assess the achievements of eco-civilization in Guangdong Province. Therefore, it is urgent to build a scientific and reasonable evaluation standard for eco-civilization.

A scientific eco-civilization evaluation index system should not only evaluate the level of eco-civilization, but also provide tailor-made policy advice and reference for decision-making (Yan et al., 2021) [6]. How to scientifically construct an eco-civilization index system and conduct eco-civilization assessment is a critical practical content for measuring the state of eco-civilization, assessing the performance of eco-civilization building and providing early warning of ecosystem health (Zhang et al., 2014) [7]. Therefore, a systematic, efficient and comprehensive dynamic assessment of the eco-civilization of Guangdong Province is of great significance in facilitating the progress of the further development of the eco-civilization of Guangdong Province, and will help Guangdong Province make targeted adjustments to the indicator dimensions that score weakly in the rating of the eco-civilization. The AHP (Analytical hierarchical process) approach allows for the analysis of the data collected and use quantitative and qualitative standard for joint evaluation by establishing and reckoning with the selection criteria, thus simplify and systematize complex decision-making processes (Şener et al., 2011) [8]. Meanwhile, GIS (Geographic Information System) enables rapid, intelligent and dynamic processing and analysis of data with different attribute content. Therefore, this paper uses a combination of AHP & GIS to work out which indicators ought to be given in the assessment of eco-civilization and to determine the regional spread dynamic of the state of eco-civilization development in the same region, and thus to determine the degree of ecological civilization development. The innovations in this essay are: (1) By analyzing the study of relevant literature and industry reports in depth, a system of criteria (resource conditions, eco-environment, economic benefits, social development criteria and their sub-criteria) is established to further evaluate the establishment of eco-civilization. (2) GIS-AHP is used to calculate the weights of relevant indicators and visualise them in ecological civilization building levels, so as to visualise the differences in ecological civilization building levels among different cities, and to facilitate the government to make appropriate policies for cities with different ecological civilization building levels according to local conditions.

**Fig. 1:**
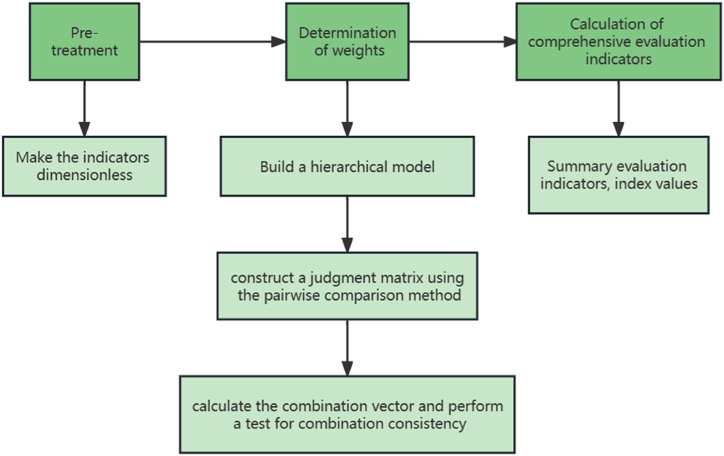
Workflow of the assessment method.

The workflow of the evaluation method in this paper is shown in Fig. 1. First, we construct ecological civilization evaluation indicators through the expert evaluation method. Then, in the stage of determining the weights of each index, we first build a hierarchical evaluation model, construct a judgement matrix by using the pairwise comparison method, and then carry out a combined consistency test by calculating the combined weight vector. Finally, we will calculate the comprehensive evaluation index - adding up the weights and index values of the evaluation indicators.

## 2 Literature review

As an important part of China's socialist construction and a basic development strategy, eco-civilization is a long-term plan for the well-being of the people and the fate of the nation. To build an eco-civilization is to solve problems in various fields such as economic, social, humanities, people's livelihoods and resources and the environment in economic growth, and to achieve sustainable economic and social development. An eco-civilization evaluation system can be established for the sake of incorporating the construction of eco-civilization into the practical operational level (Li, 2015) [9]. When establishing a comprehensive evaluation index system, the principles of objectivity, feasibility, comprehensiveness, simplicity and rationality must be observed in order to measure the state of eco-civilization establishment, and also so that the development of the whole economy and society can be evaluated comprehensively and objectively (Wang et al., 2015) [10]. With the in-depth research on theories and practical models related to eco-civilization in China, scholars have now proposed several sets of eco-civilization assessment index systems at the national level, provincial level, regional level，city level and county level, and we have classified and summarized these results, as shown in Table 1. Overall, the existing eco-civilization assessment index systems have provided quantitative standards for the building and development of eco-civilization, and have had a positive effect in guiding the building of eco-civilization in depth, improvement, expansion and enhancement (Yang et al., 2015) [11].

**Table 1:** Progress of research on eco-civilization evaluation index system.

Serial number	Type	Researchers	Research target
1	National level	Chen & Shi (2022) [12]	An evaluation of the dynamic characteristics and influencing factors of ecological civilization in China
2		Veisi et al. (2016) [13]	Define strategies and ethics for sustainable development of Iran's agricultural and food systems.
3		Dong (2021) [14]	Construct an eco-civilization performance evaluation systemin 30 Chinese provinces and cities
4		Wu et al. (2021) [15]	Assess the progress of eco-civilization in China's provinces
5		Chen et al. (2022) [16]	Explore the efficiency of eco-civilization in China's provinces
6		Ye et al. (2023) [17]	Analyse the degree of regional differentiation in the achievement of eco-civilization in China.
8		Cheng et al. (2018) [18]	Ecological civilization evaluation in China and seven small and medium-sized cities in Hubei Province.
9		Yang& Huang(2020) [19]	Measure the level of urban eco-civilization in China based on data from 285 cities at prefecture level and above from 2003 to 2016
10	Provincial level	Duan &Wang (2022) [20]	Cross-provincial eco-civilization in China was assessed
11		Wang et al. (2021) [21]	An evaluation of urban ecological civilization and identification of its key barriers
12		Wang et al. (2015) [10]	A comprehensive evaluation and cluster analysis of the development status of ecological civilization
14		Dong et al. (2020) [22]	Improve the provincial eco-civilization evaluation system
15		Yue (2022) [23]	Evaluate the performance of each province in building ecological civilization
17	Regional level	Qi et al. (2018) [24]	Build a regional eco-civilization assessment index system
18		Zheng et al. (2021) [25]	Measure the level of eco-civilization
20		Mi et al. (2018) [26]	Carve out the pattern of path dependence and evolution of ecological civilization construction in the Yangtze River Economic Belt, 2003–2015
21		Boori et al. (2021) [27]	Regional ecological environmental quality assessment
23		Hu (2022) [28]	Evaluation of the level of development of eco-civilization in coastal areas of Jiangsu
24		Checon et al. (2023) [29]	Evaluate the effectiveness of human stress and natural variation gradients on the ecological quality of 90 beach locations.
		Von et al. (2023) [30]	Develop an ecological production framework for ecological evaluation of urban stream pressure sources, conditions, human use, and preferences in the Piedmont Ecological Area of the United States
25	City level	Zhang et al. (2019) [31]	An assessment of the development of eco-civilization
26		Zhang et al. (2022) [32]	The state and changes of eco-civilization
27		Zhang et al. (2018) [33]	Construct an eco-civilizational urban resource and environmental carrying capacity index system
28		Wang et al. (2020) [34]	Constructing a Comprehensive Evaluation Model for Sustainable Development in Jinan City
29		Li & Qu (2018) [35]	Ecological civilization status of four sub-provincial cities
30		Tian et al. (2022) [36]	A dynamic research framework for urban ecological safety assessment and prediction
31		Javadian et al. (2011) [37]	Analysis of the environmental suitability
32		Aizizi et al. (2023) [38]	Calculate remote sensing ecological index and explore its main influencing factors
33		Guha et al. (2017) [39]	Dynamic analysis and ecological evaluation of urban heat island in Raipur, India
34		Navidi et al. (2023) [40]	Assess the ecological potential of agricultural land in Bastam, Semnan Province, Iran.
35	County level	Jokar & Masoudi (2023) [41]	Evaluate the ecological capacity of different land uses in Jarom County, Iran using the new EMOLUP model

Based on the above studies, in general, scholars are using the composite weighted index method (Wu et al., 2021) [42], hierarchical analysis (Javadian et al., 2011; Chen & Shi, 2022; Zhang et al., 2018; Li & Qu, 2018; Veisi et al., 2016) [12,13,33,35,37], entropy weighting method (Yue, 2022; Wang et al., 2020; Cheng et al., 2018) [18,23,34], spatio-temporal factor analysis (STFA) (Dong et al., 2020) [22], TOPSIS method (Wang, 2021; Zheng et al., 2021) [21,25], BP neural network (Hu, 2022) [28] and other methods to construct an evaluation index system for the creation of ecological civilization from multiple perspectives and calculate the degree of eco-civilization establishment in each region in order to evaluate eco-civilization construction, providing quantitative criteria for the construction and development of eco-civilization, which has had a positive effect in guiding the construction of eco-civilization to continuously deepen, improve, expand and enhance (Yang et al., 2015) [11]. However, there are also certain shortcomings: (1) In the selection of indicators, most scholars tend to consider nature, society and economy in isolation, and tend to focus on one aspect in the selection of indicators, while the proportion of evaluation indicators for the evaluation of other aspects has yet to be strengthened. (2) Most studies are static evaluations: at present, there are few dynamic studies on the construction of eco-civilization. For the evaluation of the building of eco-civilization at the provincial level, scholars generally take one year or a span of years as the study period, and it is not common to conduct continuous dynamic visual evaluation of the prefecture-level cities in the province, making it difficult to make horizontal comparison and lack of global path vision for the building state of eco-civilization in cities in the same province. (3) The current research method is relatively basic and single. At present, most of the available literatures on the assessment of the eco-civilization establishment degree are on the basis of a specific method to assess the level of eco-civilization establishment of the subject under study.

The hierarchical analysis method (AHP method) is a combined qualitative and quantitative decision analysis method for solving complex problems with multiple objectives. This method is particularly suitable for the evaluation of systems with multiple objectives, criteria, periods, etc. (Vaidya & Kumar, 2006) [43]. At the same time, geographic information systems (GIS) enables fast, efficient and flexible processing and analysis of data with various elements of attributes (Günen, 2021) [44]. In this paper, the combination of AHP & GIS can better determine which criteria should be considered for the assessment of eco-civilization establishment, determine the dynamic spatial distribution of the state of eco-civilization establishment in the same region, and thus identify the degree of development of eco-civilization. Research using a composite method combining hierarchical analysis (AHP) and geographic information systems (GIS) for dynamic level evaluation is still relatively rare.

Therefore, the possible marginal contributions of this study are mainly in three aspects: firstly, in the selection of indicators: this study combines previous studies and takes into account “innate” stock factors such as natural resources and “acquired” factors such as ecological environment, economic efficiency and social construction. The study also takes into account the actual geographical and human conditions of Guangdong Province and the availability of data, and establishes a four-indicator evaluation system of “resource-environment-economy-society” for the building level of eco-civilization in Guangdong Province. Secondly, in terms of research time series, this paper conducts a dynamic evaluation of the level of eco-civilization building, and selects continuous time panel data from 2017 to 2021 for each city in Guangdong, and conducts long-term dynamic monitoring of the achievements of eco-civilization establishment in Guangdong Province, and makes effective suggestions for the establishment of eco-civilization in Guangdong. Third, in terms of research methodology: This paper firstly adopts hierarchical analysis to calculate the weight of each evaluation index, and then uses GIS technology to present the ecological civilization scores of each city in Guangdong Province in a hierarchical visualisation in order to reveal the geographical dispersion of the scale of ecological civilization construction in Guangdong Province, so that the ecological civilization construction achievements of each city in Guangdong Province will be revealed in an intuitive and visual manner, which is convenient for the readers to find out the problems in a more intuitive way.

## 3 Constructing an ecological civilization evaluation index system in Guangdong Province

Guangdong Province, as the leader in reform and opening up, has an important role as a model for the evaluation system and construction reform of eco-civilization. For the sake of design an eco-civilization assessment system with relevance and use value based on the actual situation of the level of eco-civilization achievement in Guangdong Province, certain, more scientific design principles need to be followed. From previous research, it is clear that the development of ecosystems is based on the interaction of environmental, resource, social and economic carrying capacities (Graymore et al., 2010; Kondyli, 2010) [45,46]. Therefore, on the basis of the existing system, this paper follows the principles of operability, practicality, importance and systematicity, and constructs the system from four aspects: resource status, eco-environment, economic benefits and social development (Table 2)."+" indicates that the indicator has a positive effect on the overall score of the dynamic evaluation of ecological civilization, and "-" indicates that the indicator has a negative effect on the overall score of the dynamic evaluation of ecological civilization.

**Table 2:** Guangdong Province eco-civilization evaluation index system.

Target layer	Standard Layer	Indicator layer	Calculation method	Properties	The significance of the assessment
The overall goal of the establishment of eco-civilization in Guangdong	Resource conditions	Commonly used arable land area (mu)	Statistical indicators	+	Assessment of the status of the output capacity of arable land, water, forest and energy resources
		Forest cover (%)	Statistical indicators	+	
		Water resources per capita (m^3^/person)	Statistical indicators	+	
		Total energy consumption (million tons of standard coal)	Statistical indicators	–	
		Total output value of agriculture, forestry, animal husbandry and fishery (billion yuan)	Statistical indicators	+	
	Ecology	Total apparent CO2 emissions (million tons)	Statistical indicators	–	Assessment of atmospheric environmental quality indicators
		Urban green space area (hectares)	Statistical indicators	+	Assessment of environmental construction indicators
		Harmless disposal rate of domestic waste (%)	Statistical indicators	+	Assessment of pollution control indicators
		Urban sewage treatment rate (%)	Statistical indicators	+	
		Green total factor productivity (%)	Urban total factor productivity growth is measured by the Malmquist productivity index, an over-efficient SBM model that integrates undesired outputs, in a global covariate data envelopment analysis framework	+	Assessment of production environment indicators
	Economic efficiency	Per capita disposable income of residents (10,000 yuan)	Statistical indicators	+	Evaluate the overall economic level, per capita income, and living standards of residents
		Gross regional product (billion yuan)	Statistical indicators	+	
		Resident gini index	Statistical indicators	–	
		Engel's index	Total food expenditure/Total household or individual consumption expenditure	–	
	Social Development	Number of urban public transportation vehicles (vehicles)	Statistical indicators	+	Assess the basic security indicators of residents' life and well-being
		Number of people receiving insurance benefits from basic pension insurance (10,000)	Statistical indicators	+	
		Number of people receiving insurance benefits from social health insurance (10,000)	Statistical indicators	+	
		Annual average population of resident population (10,000 people)	Statistical indicators	–	
		Ecological attention	Government work report ecological environment keyword statistics	+	Evaluate the government's attention to and implementation of ecological environment and residents' well-being and livelihood security
		Number of health institutions	Statistical indicators	+	

As far as the resource situation is concerned, the diversity of resources provides a solid foundation for the construction of eco-civilization regard to resources and environment. Land resources, water resources and forest resources are important resources, and the total energy consumption and the total output value of agriculture, forestry, animal husbandry and fishery can clearly reflect the capacity of resource utilization. In recent years, with the continuous expansion of urban scale and the continuous consumption of land resources, the effect of sustainable use of land resources has been weakened to a certain extent, making the sustainable development of society threatened to a greater extent. At the same time, water resources, water ecology, water environment and water disasters are frequent (Yuan & Zheng, 2022) [47], and water resources play an extremely important role in human production and life, so it is important to monitor and examine water resources. Today, the threat of severe weather phenomena such as climate change impacts, fires, insect and plant invasions, diseases, storms, floods, temperature changes or droughts, and increased economic and human activities have put forests around the world under a great deal of pressure (Cyfert & Dyduch, 2022) [48], and forest resources have always played a pivotal role in advancing the course of human civilization, and their It is important to protect them. Energy as a raw material for power has always been an essential element in driving human civilization (Hoho et al., 2015) [49]. At the same time, the measurement of energy consumption is extremely important today. The total output value of agriculture, forestry, animal husbandry and fishery is also an important indicator of resource utilization capacity. Therefore, this paper evaluates the resource situation using five indicators: commonly used arable land area, per capita water resources, forest coverage, total energy consumption and total output value of agriculture, forestry, animal husbandry and fishery.

In terms of ecological environment, eco-environmental civilization is a visual indicator of the state of eco-civilization and is the aspect that most directly reflects its effectiveness. In terms of its role, eco-environmental civilization is fundamental. Judgement of eco-environmental civilization should take into account the endogenous relationship and coupled coordination mechanism of the coordinated development of economic, eco-environmental and health systems (Hou et al., 2022) [50]. Therefore, this paper draws on the opinions of relevant experts and selects total apparent carbon dioxide emissions, urban green space, urban household waste disposal rate, green total factor productivity and urban wastewater treatment rate as evaluation indicators.

In terms of economic efficiency, a healthy economy is the foundation of ecological civilization. The economy provides the material and financial basis for the construction of ecological civilization with its material regeneration function (Hoho et al., 2015) [49]. Therefore, in this paper, quantifiable indicators that can be used to comprehensively measure economic efficiency from the perspective of the level of economic development and the fairness of income distribution are selected, namely disposable income per inhabitant, gross regional product, Gini index, and Engel index.

The stable development of society facilitates the building of an eco-civilization at the level of social development. Services and infrastructure are one of the key indicators for achieving optimal carrying capacity (George & Kini, 2016) [51]. Therefore, in this paper, the number of vehicles operating in urban public transport, the number of people receiving insurance benefits under basic pension insurance, the number of people receiving insurance benefits under social health insurance, the average annual population of the resident population, ecological attention, and the number of health institutions are selected from the government's concern, execution, and residents' well-being and security, respectively.

## 4 Data sources and assessment methods

### 4.1 Data source and pre-processing

To evaluate the state of eco-civilization establishment in Guangdong Province and its 21 prefectural cities from 2017 to 2021. The data were mainly obtained from the statistical yearbook of Guangdong Provincial Bureau of Statistics (http://stats.gd.gov.cn/gdtjnj/). At the same time, the national level of eco-civilization in the current year is used as a target for reference, and the observed indicators are indexed. The specific formula (1) (2) is as follows:

(1) $$\text{Positive}\;\text{dimension:}y_{\text{ij}}=\left(x_{\text{ij}}/B_{ij}\right)\times 100$$

(2) $$\text{Negative}\;\text{dimension:}y_{\text{ij}}=\left(B_{ij}/x_{ij}\right)\times 100$$

Where $x_{\text{ij}}$ is the j-th indicator and observation value of the i-th evaluation object, $B_{ij}$ is the national average of the i-th indicator and the j-th evaluation object, and is the unfactored value of the i-th indicator and the j-th evaluation object. Taking 2021 as an example, the evaluation value of the level of eco-civilization establishment in Guangdong Province by the dimensionless method as indicated in Table 3. We suppose that the average for China stands at 100 points.

**Table 3:** Evaluation of eco-civilization indicators in Guangdong Province in 2021.

City	Guangzhou	Shenzhen	Zhuhai	Shantou	Foshan	Shaoguan	Heyuan	Meizhou	Huizhou	Shanwei	Dongguan
Commonly used arable land area (mu)	6.70	0.30	1.14	15.73	2.12	27.60	30.48	42.03	25.68	18.73	0.43
Forest cover (%)	180.87	171.30	140.00	162.70	93.04	323.48	317.30	323.91	267.83	213.04	158.26
Per capita water resources (m^3^/person)	37.54	5.95	11.55	10.47	24.18	300.79	221.13	126.73	87.47	112.73	12.38
Total energy consumption tons of standard coal (million tons)	13.35	94.62	10.40	64.73	59.47	14.56	40.12	63.67	112.95	67.10	32.48
Total output value of agriculture, forestry, animal husbandry and fishery in each city (billion yuan)	108.14	9.23	19.72	47.05	78.58	71.84	47.95	77.46	77.23	55.50	10.64
Total apparent CO2 emissions	26.47	51.10	111.01	78.82	37.61	127.90	157.04	110.71	45.42	154.94	40.67
Urban green space area	457.04	864.38	137.49	104.30	63.08	42.29	14.30	23.82	101.13	10.39	749.92
Harmless treatment rate of urban domestic waste (%)	100.30	100.30	100.30	100.30	100.30	100.30	86.66	100.30	100.30	100.30	100.30
Green total factor productivity	97.32	100.30	96.33	95.33	100.30	102.28	100.30	98.31	105.26	98.31	104.27
Urban sewage treatment rate (%)	99.81	100.42	101.75	100.83	103.48	104.10	99.81	101.13	100.11	98.58	99.30
Per capita disposable income of residents (10,000 yuan)	196.32	201.84	174.90	88.23	175.78	86.07	70.16	74.67	123.51	78.13	177.00
Gross regional product (billion yuan)	719.80	781.83	98.97	74.70	309.94	39.62	32.48	33.35	126.90	32.84	276.77
Resident gini index	299.48	1647.15	1098.10	253.41	274.53	91.51	70.09	137.26	109.81	173.38	3294.30
Engel's index	77.62	79.32	158.25	122.56	111.25	123.48	114.39	112.03	152.34	104.82	107.95
Number of public transportation	635.38	699.47	101.52	84.62	284.45	32.95	16.29	84.12	126.24	58.08	260.39
Number of pensioners (10,000)	39.76	0.34	3.48	62.76	13.97	29.34	37.48	48.17	31.70	27.22	1.58
Number of people with social health insurance	107.99	69.85	14.59	94.10	50.84	50.30	57.28	85.86	54.33	55.54	141.51
Ecological attention	106.12	71.43	90.82	123.47	121.43	140.82	163.27	122.45	36.73	127.55	114.29
Number of health institutions	166.54	167.40	29.96	56.23	72.53	61.44	60.61	84.42	101.60	46.49	98.37
Average resident population (10,000)	25.63	27.27	195.45	87.17	50.15	168.56	169.70	124.35	79.48	179.43	45.75

City	Zhongshan	Jiangmen	Yangjiang	Zhanjiang	Maoming	Zhaoqing	Qingyuan	Chaozhou	Jieyang	Yunfu
Commonly used arable land area (mu)	0.65	42.66	27.65	64.41	57.46	45.73	34.48	9.69	29.92	23.52
Forest cover (%)	100.43	196.09	249.57	101.96	242.61	307.39	302.61	259.52	221.26	296.09
Per capita water resources (m^3^/person)	25.80	108.65	212.33	125.45	89.93	177.10	321.43	53.32	58.92	9.82
Total energy consumption tons of standard coal (million tons)	134.29	240.10	145.41	91.87	57.58	17.44	47.86	1326.05	88.40	184.68
Total output value of agriculture, forestry, animal husbandry and fishery in each city (billion yuan)	27.87	108.08	81.14	207.94	207.84	143.86	101.31	39.12	65.98	69.06
Total apparent CO2 emissions	70.44	55.50	189.72	85.99	109.38	95.58	82.46	154.00	87.45	186.80
Urban green space area	47.99	60.82	109.55	40.98	44.61	121.08	48.23	27.79	96.94	13.23
Harmless treatment rate of urban domestic waste (%)	100.30	100.30	100.30	79.34	100.30	100.30	100.30	100.30	100.30	100.30
Green total factor productivity	99.30	98.31	95.33	95.33	102.28	96.33	96.33	99.30	101.29	96.33
Urban sewage treatment rate (%)	91.53	99.60	121.97	98.78	105.53	98.78	95.82	109.00	99.09	98.99
Per capita disposable income of residents (10,000 yuan)	164.96	105.61	83.10	78.76	76.15	86.59	81.88	71.46	67.75	70.12
Gross regional product (billion yuan)	90.92	91.82	38.65	90.76	94.29	67.56	51.18	31.74	57.76	29.04
Resident gini index	3294.30	113.60	99.83	89.04	82.36	71.62	67.23	143.23	149.74	156.87
Engel's index	136.97	141.74	141.74	164.65	101.88	119.85	116.01	96.17	90.81	89.81
Number of public transportation	105.92	72.23	20.24	44.88	49.98	41.88	44.71	16.62	27.97	17.94
Number of pensioners (10,000)	0.23	43.11	35.57	80.69	77.03	46.61	49.29	31.20	70.03	34.09
Number of people with social health insurance	24.66	52.92	51.44	138.14	131.77	72.30	71.65	48.31	118.61	50.01
Ecological attention	124.49	90.82	91.84	109.18	104.08	81.63	89.80	86.73	111.22	112.24
Number of health institutions	32.80	49.76	51.85	104.35	112.63	92.09	75.11	64.39	91.55	39.99
Average resident population (10,000)	107.93	99.71	183.96	68.57	77.51	116.74	121.05	187.26	85.83	201.44

**Table 4:** Weighting of eco-civilization evaluation indicators in Guangdong Province.

Tier 1 indicator layer	Weights	Secondary indicator layer	Weights
Resources level	0.3052	Commonly used arable land area (ha)	0.0757
		Forest cover	0.1237
		Water resources per capita (m3/person)	0.0484
		Total energy consumption (million tons of standard coal)	0.0206
		Total output value of agriculture, forestry, animal husbandry and fishery (billion yuan)	0.0368
Environmental protection	0.4505	Total apparent CO2 emissions (million tons)	0.0471
		Urban green space area (hectares)	0.0920
		Harmless disposal rate of domestic waste (%)	0.1279
		Green total factor productivity (%)	0.0964
		Ecological attention	0.0383
		Sewage treatment rate	0.0488
Economic development	0.1072	Per capita disposable income of residents (10,000 yuan)	0.0449
		Gross regional product (billion yuan)	0.0233
		Resident gini index	0.0303
		Engel coefficient	0.0087
Social construction	0.1371	Urban public transport operating vehicles, boats (vehicles, ships)	0.0427
		Number of people receiving insurance benefits from basic pension insurance	0.0205
		Number of people receiving insurance benefits from social health insurance	0.0482
		Number of health institutions	0.0078
		Number of resident population	0.0179

### 4.2 Evaluation methodology

In this study, the Delphi method was used to consult experts in order to assess the weights of resource conditions, ecological environment, economic benefits and social development among the four dimensions of ecological civilization in Guangdong Province, and the questionnaire was subjected to 2–3 rounds of expert consultation, which eventually resulted in more consistent answers. A credibility test was also conducted, and α was 0.965, indicating good credibility. The hierarchical analysis method was used to calculate the weights of each indicator and construct a comprehensive evaluation index to evaluate the results of ecological civilization construction in Guangdong Province from 2017 to 2021.

(1) Determine the weights. Firstly, a hierarchical model was established based on the above indicators. Secondly, on the basis of expert scoring, the judgment matrix was constructed using the pairwise comparison method and the 1–9 comparison method; then the weight vector was calculated and a consistency test was performed. Finally, we calculated the combined weight vector and carried out a comprehensive consistency test. Based on the conclusions of the 17 experts, we obtained the weightings of each eco-civilization indicator in Guangdong (Table 4).

(2) Calculate the comprehensive evaluation index. Based on the index weights calculated by the AHP method, a comprehensive evaluation index of ecological civilization is constructed. Firstly, the evaluation index values in the indicator layer that have been dimensionless processed are summed up depending on the respective target weights, the corresponding values of the assessment indexes are then available within the criteria layer are obtained in the standard layer. Secondly, the values of the indicators were aggregated for the period 2017–2021 according to the respective target weights to arrive at the assessment results of the establishment of Guang Dong Province's ecological civilization. The specific formulas (3) (4) are shown below:

(3) $$e_{j}=\sum_{i=1}^{n}y_{i}w_{i}$$

(4) $$E=\sum_{j=1}^{n}e_{j}w_{j}$$

Among them, $y_{i}$ is the dimensionless value of the i-th indicator, $e_{j}$ is the assessed value of the j-th standard, E denotes indicators for assessing the eco-civilized degree, $w_{i}$ & $w_{j}$ are the weightings of the i-th index and the j-th parameter separately.

## 5 Analysis of research results

Based on the approach to evaluation described the above, we have obtained the 2017–2021 assessment figures for the degree of building eco-civilization in Guangdong Province and prefectural cities. (Table 5).

**Table 5:** Evaluation scores of eco-civilization indicators in Guangdong Province, 2017–2021.

Resources level	Guangzhou	Shenzhen	Zhuhai	Shantou	Foshan	Shaoguan	Heyuan	Meizhou	Huizhou	Shanwei	Dongguan
2017	28.56	26.72	19.08	18.99	18.46	59.43	55.62	54.76	45.97	39.67	22.37
2018	28.69	24.55	19.16	19.04	16.87	59.57	55.20	54.07	44.85	38.21	21.91
2019	28.54	24.32	19.09	18.92	16.41	59.10	54.68	53.47	44.60	37.89	21.82
2020	31.23	40.05	20.68	29.83	27.11	61.76	61.43	64.61	63.67	48.98	26.79
2021	28.95	23.79	18.90	24.89	16.96	59.61	54.85	53.55	44.48	36.65	21.27
Environment level	Guangzhou	Shenzhen	Zhuhai	Shantou	Foshan	Shaoguan	Heyuan	Meizhou	Huizhou	Shanwei	Dongguan
2017	162.80	128.09	122.16	48.50	44.52	39.68	43.87	38.59	41.00	43.76	41.20
2018	159.53	129.89	120.26	53.32	47.77	41.12	45.31	42.08	45.27	51.05	43.07
2019	156.00	122.66	115.92	49.23	44.31	36.68	43.68	39.49	41.56	47.72	41.17
2020	148.74	113.28	109.42	44.99	40.50	35.21	35.15	35.00	37.05	42.77	39.35
2021	148.01	109.33	44.96	40.25	35.12	37.68	34.34	34.65	39.30	35.37	98.63
Economy level	Guangzhou	Shenzhen	Zhuhai	Shantou	Foshan	Shaoguan	Heyuan	Meizhou	Huizhou	Shanwei	Dongguan
2017	35.72	62.89	45.72	13.79	23.96	8.54	6.81	9.59	12.95	10.72	118.90
2018	35.82	79.73	45.67	14.11	24.12	8.45	6.87	9.42	12.96	10.64	118.76
2019	35.21	80.38	45.97	14.74	24.90	8.57	6.96	9.40	13.02	10.39	119.21
2020	35.81	80.34	115.40	15.45	24.73	8.58	7.05	9.22	13.11	10.80	119.23
2021	35.34	77.88	44.81	14.45	24.40	8.63	7.03	9.26	13.15	10.44	115.15
Social level	Guangzhou	Shenzhen	Zhuhai	Shantou	Foshan	Shaoguan	Heyuan	Meizhou	Huizhou	Shanwei	Dongguan
2017	72.40	75.43	17.73	20.72	35.98	16.83	15.96	21.19	26.45	17.87	37.57
2018	53.49	58.06	15.68	18.56	28.92	13.83	13.77	19.70	21.69	16.66	32.42
2019	45.93	45.63	14.08	17.51	24.35	14.45	15.15	18.92	17.27	15.14	26.69
2020	45.95	42.82	18.31	18.73	22.69	13.81	16.10	18.76	14.57	14.01	24.39
2021	38.97	37.77	12.32	16.16	21.00	13.32	13.99	16.29	12.28	14.17	23.93

Resources level	Zhongshan	Jiangmen	Yangjiang	Zhanjiang	Maoming	Zhaoqing	Qingyuan	Chaozhou	Jieyang	Yunfu
2017	21.73	72.48	57.82	33.43	48.35	55.33	61.29	39.15	37.15	50.09
2018	18.04	41.70	50.47	34.19	47.66	55.70	60.58	38.19	36.50	45.26
2019	17.60	41.59	49.54	33.34	47.81	55.36	59.92	38.35	36.34	45.05
2020	40.39	97.99	74.45	48.87	57.93	58.58	68.15	291.50	51.94	76.47
2021	17.51	41.67	49.22	33.10	47.55	55.71	60.31	64.17	36.74	45.23
Environment level	Zhongshan	Jiangmen	Yangjiang	Zhanjiang	Maoming	Zhaoqing	Qingyuan	Chaozhou	Jieyang	Yunfu
2017	39.17	43.38	42.51	42.22	36.86	108.55	38.07	40.94	40.96	44.83
2018	44.06	49.89	48.86	49.13	40.68	107.82	40.83	50.29	45.35	52.39
2019	39.47	43.48	43.49	43.24	37.72	104.10	38.08	44.56	41.68	45.93
2020	34.60	35.59	36.30	44.23	35.52	98.89	35.14	37.13	40.58	37.33
2021	34.60	35.38	46.98	31.98	37.09	42.58	35.11	37.53	40.47	36.96
Economy level	Zhongshan	Jiangmen	Yangjiang	Zhanjiang	Maoming	Zhaoqing	Qingyuan	Chaozhou	Jieyang	Yunfu
2017	115.35	10.99	8.86	9.51	9.11	8.77	7.78	9.30	10.02	9.45
2018	115.14	11.15	8.84	9.55	9.08	8.63	7.67	9.38	10.05	9.59
2019	114.56	11.51	8.86	9.67	9.06	8.60	7.79	9.34	10.03	9.37
2020	114.56	11.61	9.04	10.07	9.22	8.64	7.83	9.51	10.16	9.05
2021	110.53	11.56	8.89	9.78	9.00	8.67	7.92	9.12	9.71	9.36
Social level	Zhongshan	Jiangmen	Yangjiang	Zhanjiang	Maoming	Zhaoqing	Qingyuan	Chaozhou	Jieyang	Yunfu
2017	19.21	20.40	14.99	23.05	22.18	18.13	18.05	13.68	21.89	14.25
2018	18.99	16.16	12.84	19.92	18.43	14.93	15.93	12.35	17.67	12.73
2019	16.47	14.75	13.19	19.53	18.64	14.52	14.80	13.38	18.67	12.21
2020	15.87	14.80	14.42	16.79	20.27	15.54	15.26	12.19	18.49	11.22
2021	12.67	12.17	11.29	16.45	16.32	12.16	12.56	10.85	14.86	12.09

**Fig. 2:**
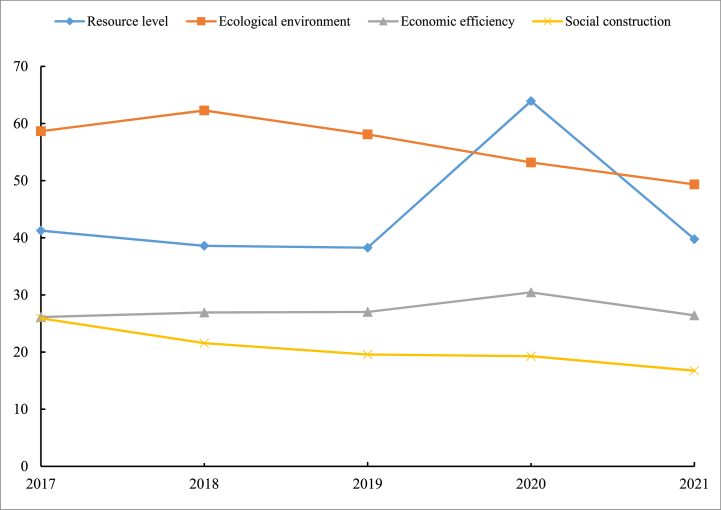
Guangdong Province eco-civilization evaluation results.

It is clear from Table 6 that the state of Guangdong Province and of its 21 municipalities at the prefecture level are generally higher than the national average in terms of eco-civilization from 2017 to 2021. From Fig. 2, It may be observed that the eco-civilization of Guangdong shows a fluctuating downward trend from 2017 to 2021. The highest level appears in 2020.

**Table 6:** Comparison of eco-civilization by cities in Guangdong Province with the provincial and national averages.

Year	Number of above national average	Percentage of	Higher than the average number of Guangdong Province	Percentage of	Below the average number of Guangdong Province	Percentage of
2017	21	100 %	6	28.57 %	15	71.43 %
2018	21	100 %	6	28.57 %	15	71.43 %
2019	21	100 %	6	28.57 %	15	71.43 %
2020	21	100 %	7	33.30 %	14	66.70 %
2021	17	81 %	4	19.00 %	17	81 %

Of the 21 municipalities at prefecture level, those with an eco-civilization status above the provincial average are relatively constant, but there is a tendency to decrease. Only four municipalities, or 19 % of the provincial total, will be represented in 2021. Depending on the evaluation results, these four cities are:Dongguan, Shenzhen, Zhongshan, Guangzhou, these cities in the forefront of Guangdong Province scored much higher than other cities, pulling up the average score level of Guangdong Province, making as many as 17 other cities in Guangdong Province below the provincial average, but most cities are not much different from the average level of eco-civilization in Guangdong Province. During the ecological civilization study period, the state of eco-civilization building in different cities changed differently (as shown in Table 7). The levels of Shenzhen, Dongguan, and Chaozhou showed a fluctuating upward trend while the levels of Guangzhou, Heyuan, and Zhaoqing continued to decline, and the rest of the cities showed a fluctuating downward trend.

**Table 7:** Dynamic changes in urban eco-civilization in Guangdong Province.

Trends	Number of cities	%	City name
Continuously rising	0	0	None
Rising volatility	3	14.29 %	Shenzhen, Dongguan, Chaozhou
Fluctuation decline	15	71.42 %	Zhuhai, Shantou, Foshan, Shaoguan, Meizhou, Huizhou, Shanwei, Zhongshan, Jiangmen, Yangjiang, Zhanjiang, Maoming, Qingyuan, Jieyang, Yunfu
Continuous decline	3	14.29 %	Guangzhou, Heyuan, Zhaoqing

From Table 8, it can be seen that the ecological civilization evaluation level map of Guangdong Province from 2017 to 2023 clearly shows that the number of cities at the extremely low level of ecological civilization significantly decreased in 2020, which was a year with good overall ecological performance, while cities in other years showed an overall increasing trend; The number of cities at a low level of ecological civilization has generally changed relatively steadily; The number of cities at the moderate level of ecological civilization has shown a relatively stable change in the number of cities in other years, except for a significant decrease in 2019 and 2021; The number of cities at a high level of ecological civilization generally shows a decreasing trend; The overall number of cities at a very high level of ecological civilization shows a slow increasing trend.

**Table 8:** Research on the dynamic evaluation level grading of ecological civilization in Guangdong Province from 2017 to 2021.

Year	Extremely low level	Percentage of	Low level	Percentage of	In level	Percentage of	High level	Percentage of	Extremely high level	Percentage of
2017	4	19.05 %	3	14.29 %	8	38.10 %	5	23.81 %	1	4.76 %
2018	2	9.52 %	5	23.81 %	8	38.10 %	5	23.81 %	1	4.76 %
2019	6	28.57 %	6	28.57 %	4	19.05 %	4	19.05 %	1	4.76 %
2020	1	4.76 %	2	9.52 %	11	52.38 %	5	23.81 %	2	9.52 %
2021	9	42.86 %	4	19.00 %	4	19.00 %	2	9.52 %	2	9.52 %

**Fig. 3:**
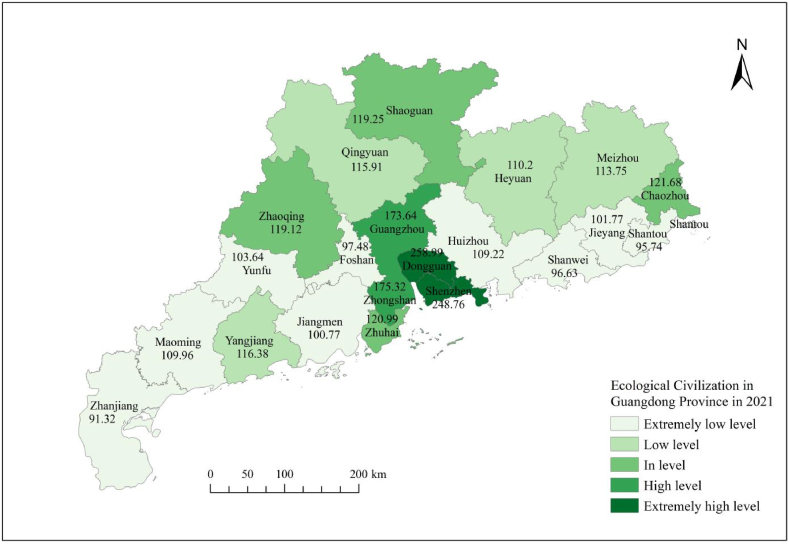
Guangdong Province 2021 ecological civilization evaluation.

**Fig. 4:**
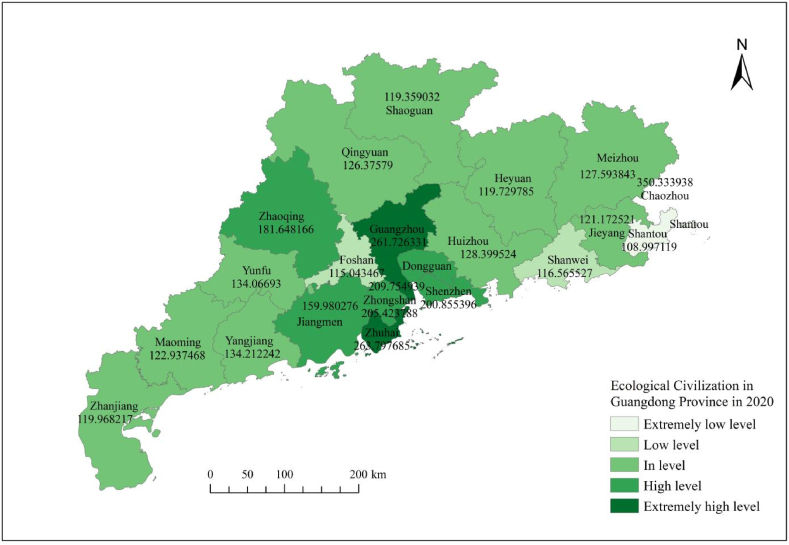
Guangdong Province 2020 ecological civilization evaluation.

**Fig. 5:**
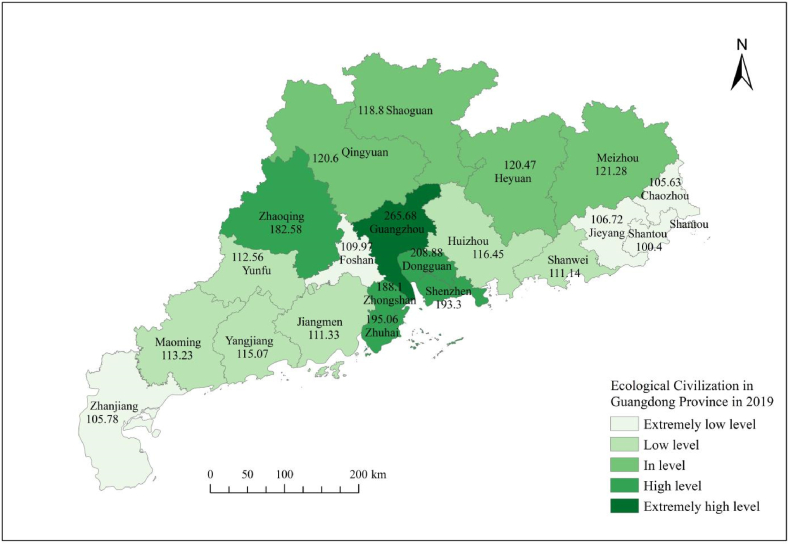
Guangdong Province 2019 ecological civilization evaluation.

**Fig. 6:**
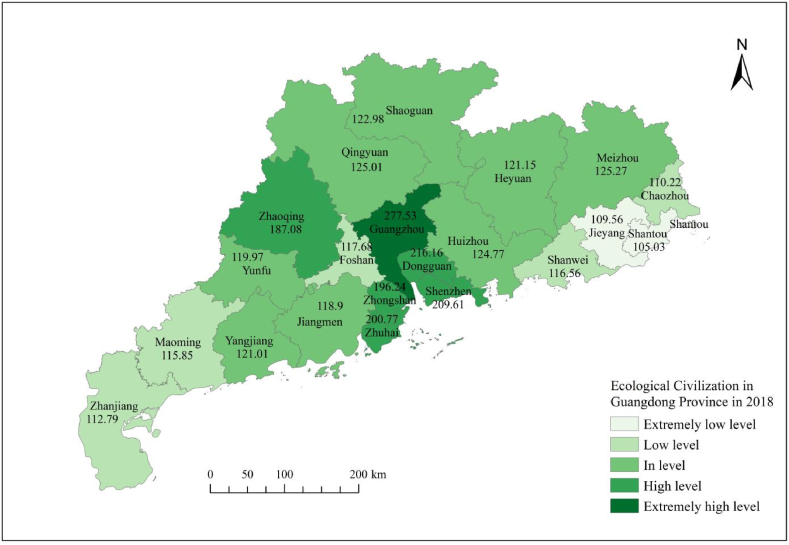
Guangdong Province 2018 ecological civilization evaluation.

**Fig. 7:**
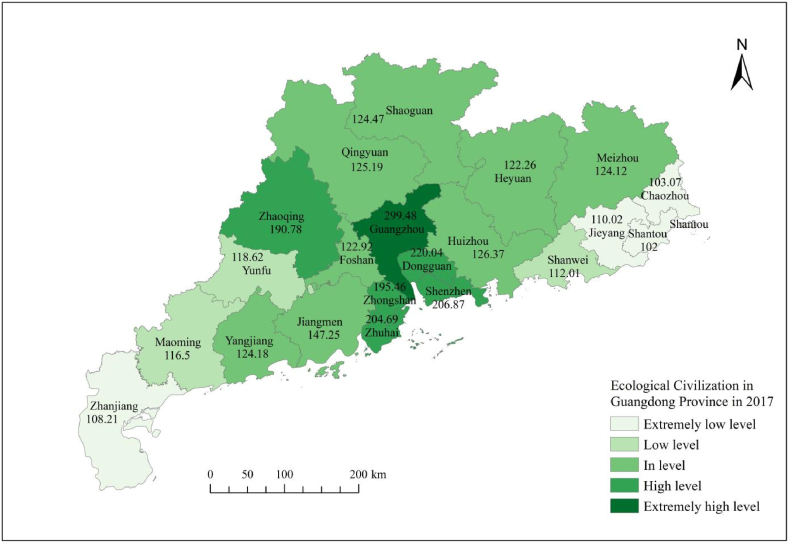
Guangdong Province 2017 ecological civilization evaluation.

**Fig. 8:**
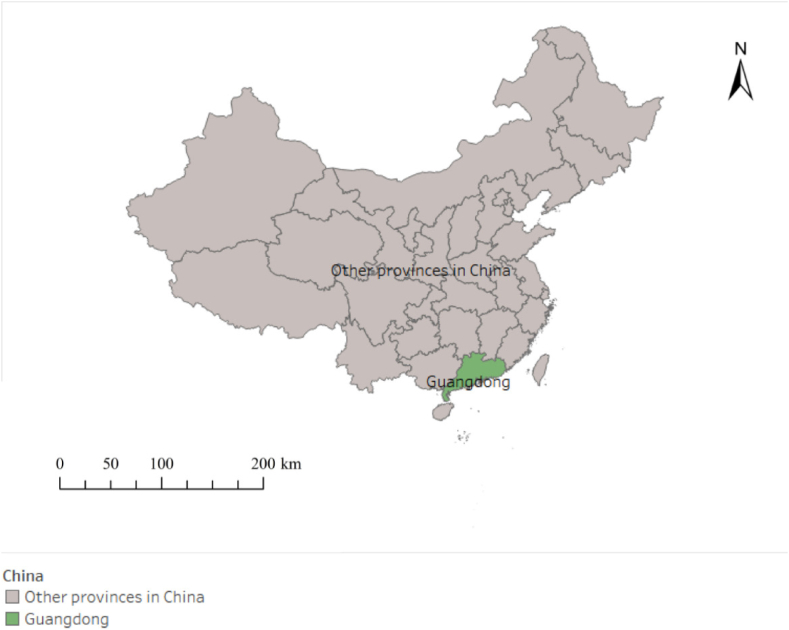
Map of Guangdong Province in relation to China.

Fig. 3, Fig. 4, Fig. 5, Fig. 6, Fig. 7 shows the spatial distribution of eco-civilization establishment state and its assessment in Guangdong Province from 2017 to 2021. It can be seen that the cities with higher eco-civilization establishment level in Guangdong are mostly concentrated in the PRD region (Pearl River Delta region), followed by northern Guangdong, while the eco-civilization building degree in eastern Guangdong and western Guangdong is more backward, but there is a trend of fluctuation and improvement. Fig. 8 shows a map of Guangdong Province in relation to China, so that readers can easily identify our study area.

Based on the preceding discussion, it can be seen that while some cities in Guangdong have improved their standards of eco-civilization, the overall degree of eco-civilization establishment in most cities still shows a downward trend, which has led to a decline in the Guangdong Province's general standard in latest decades.

## 6 City evaluation results

Based on the above analysis and the number of dimensional disadvantages of each city, the 21 cities in Guangdong Province can be roughly classified into the following three types: one-dimensional disadvantage, two-dimensional disadvantage and multidimensional disadvantage.

### 6.1 Unidimensional weaknesses

Based on the above analysis and the number of dimensional disadvantages associated with the various cities, the 21 cities in Guangdong Province can be broadly classified under three categories as follows: one-dimensional disadvantage, two-dimensional disadvantage and multidimensional disadvantage.

Guangzhou, Shenzhen and Zhuhai, as the core cities of the PRD, are far behind the province's average in the natural resource evaluation dimension, although they are far above the province's average in the overall level of eco-civilization. The main reasons for this are that Guangzhou, Shenzhen and Zhuhai are located in the alluvial plains of the PRD region, where there are fewer forest resources, and because of their developed economies and large populations, they consume resources at a faster rate and share fewer resources per capita. Not only that, compared with other dimensions, due to the rigidity of changes in natural factors (Huang & Yu, 2014) [52], the dimension of resource endowment is more confined by natural resource stocks and capacity for development, and has little impact on the comprehensive degree of eco-civilization in a short period of time, indicating that such cities have limited potential to promote the establishment of eco-civilization and should focus more on maintaining the advantages of other dimensions.

### 6.2 Weaknesses of two dimensions

The inferiority of this type of city is mainly evident in two areas and illustrates that the general degree of establishment of eco-civilization in this type of city needs to be harmonized in two areas in order to upgrade the entire standard. Based on the actual circumstances of the Guangdong, the main disadvantage is resource conditions - environmental protection.

In terms of resource conditions - environmental protection disadvantages, the following cities are the main ones: Dongguan and Zhongshan. These two cities are at the forefront of Guangdong Province in terms of economic efficiency and social development, probably because the PRD region, as one of the most dynamic economic centers in Guangdong and even in China, occupies a superior geographic location and receives sufficient resources from economic and social sources, thus driving the growth of its overall level of eco-civilization. However, such cities need to pay attention to both resource conditions and environmental protection dimensions to realize balanced and coherent progress and the ultimate goal is to strengthen the entire ecological infrastructure.

### 6.3 The weakness of multidimensional

The existence of more than three dimensions of disadvantages in this type of cities indicates a low state of eco-civilization building. It is necessary to grasp multiple dimensions at the same time, focus on the shortcomings that need to be solved, and gradually improve the total state of eco-civilization achievement. From the actual situation in Guangdong Province, it can be divided into two categories: those with advantages in resource conditions only and those without advantages in both.

Only the cities with resource conditions are represented by Shaoguan, Heyuan, Meizhou, Huizhou, Jiangmen, Yangjiang, Maoming, Zhaoqing, Qingyuan, Yunfu, and Chaozhou. Most of these cities are located in the northern part of Guangdong and have rich forest and water resources. Such cities should rely on the advantages of the dimension of resource conditions to accelerate economic and social development and improve the comprehensive level of eco-civilization building. Although the natural resource dimension of these regions still maintains a stable and good trend during the study period of 2017–2021, there is a regressive trend in the already disadvantaged environmental protection dimension and social security dimension. Therefore, such cities should also pay great attention to the negative impact of resource development on the ecological environment, as well as the enhancement of the disadvantaged social security and economic development dimensions, in the process of improving the state of eco-civilization establishment by taking advantage of their resources.

In addition to Chaozhou, which has a better advantage in terms of resource conditions in recent years by continuously optimizing its energy production structure and relying on its better forest resources, the rest of the eastern and western regions of Guangdong, which are far away from the economic hub of Guangdong Province, such as Shantou, Shanwei, Jieyang, Zhanjiang and other cities, have a low level of eco-civilization establishment in all dimensions across the province. Such cities need to learn from the experience of relevant advanced cities according to local conditions and take timely and appropriate measures to implement improvements in all areas and gradually raise the comprehensive level of eco-civilization establishment.

## 7 Research conclusions and policy recommendations

### 7.1 Research findings

(1) The total level of eco-civilization establishment in Guangdong Province is higher than the national average, but the uneven development of cities in the province is remarkable

In terms of the overall score, during the period 2017–2021, the prevailing broad standard of eco-civilization in the province of Guangdong is on average 46.31 % higher than the national average (this paper uses a combination of AHP and GIS to calculate the national average, and the data sources are all from the China Statistical Yearbook), but the development of each city is uneven. During the study period of 2017–2021, an average of six cities in Guangdong Province (Guangzhou, Shenzhen, Zhuhai, Dongguan, Zhongshan, and Zhaoqing) are higher than the provincial average, and number of municipalities with high eco-civilization scores shows a decreasing trend, with as many as 15 cities below the provincial average, indicating that the degree of eco-civilization building in Guangdong Province still needs to be improved. The findings are broadly in line with most other scholars (Wang et al., 2016; Yang et al., 2015) [11, 53]. Typically, for example, Wang et al. (2015) [10] conducted a comprehensive evaluation of the ecological civilization development status of 21 prefecture-level cities in Guangdong Province in 2012 through the methods of factor analysis, stratified factor analysis and cluster analysis. The results also show that there are significant differences in the development of cities in Guangdong Province. Based on the combination of hierarchical analysis, expert evaluation method and GIS method, this paper focuses on the results of the dynamic evaluation of ecological civilization in Guangdong Province from 2017 to 2021 by visualising the calculated data through the hierarchical visualisation of the ecological civilization development level of cities in Guangdong Province.

(2) The current eco-civilization establishment in Guangdong Province shows a fluctuating downward trend, and the construction of eco-civilization is not optimistic

In terms of the overall trend, the standard of eco-civilization establishment in Guangdong Province shows a fluctuating downward trend from 2017 to 2021. From 2017 to 2019, the index dropped from 147.83 points to 139.19 points, then rose significantly to 166.84 points in 2020, before dropping sharply to 132.29 marks in 2021. This reflects the standard of ecological building during the time period studied in Guangdong Province experienced a change of falling, then rising, then falling again, and in regards to the particular city, there are different trends exhibited by different cities. Three cities showed a fluctuating upward trend, namely Shenzhen, Dongguan and Chaozhou. Among the 18 cities showing a downward trend, 15 cities fluctuated (Zhuhai, Shantou, Foshan, Shaoguan, Meizhou, Huizhou, Shanwei, Zhongshan, Jiangmen, Yangjiang, Zhanjiang, Maoming, Qingyuan, Jieyang, Yunfu), and 3 cities continued to decline (Guangzhou, Heyuan, Zhaoqing). This finding is broadly in line with other research scholars in the field of ecological civilization (Zhao and Zhang, 2023; Wang et al., 2016; Wang et al., 2015) [10, 53, 54].

(3) The overall level of eco-civilization building in the Pearl River Delta region is higher, followed by northern guangdong, while the level of eco-civilization achievement in eastern and western guangdong is lower

In terms of the overall pattern of spatial distribution, the higher and higher levels of ecological civilization development are mostly located in the Pearl River Delta (PRD) region, probably because the PRD region, as one of the most dynamic economic centers in Guangdong and even in China, occupies a superior geographical location and receives sufficient input from economic, social and environmental resources, thus driving the growth of its comprehensive ecological civilization level; while the medium and lower level regions are mostly located in the east, west and north of Guangdong, which may be due to the relatively disadvantageous geographical location and insufficient resource input, coinciding with the national uneven development strategy since the reform and opening up, resulting in a relatively low comprehensive level of ecological civilization (Wu et al., 2017) [5]. This is the same as the viewpoint of most scholars who study ecological civilization construction related. For example, Zhao and Zhang (2023) [54] used the SBM-DDF model to measure the efficiency of ecological civilization construction in Guangdong Province, and then applied the kernel density estimation method to study the time evolution trend and variability of the efficiency of ecological civilization construction in Guangdong Province, and finally came to the conclusion that the efficiency level in the Pearl River Delta (PRD) region is higher, and that in the western and northern regions of Guangdong is lower. This is generally in line with the findings of this paper.

(4) The economic development dimension has a great influence on the establishment of eco-civilization in guangdong, and the state of eco-civilization establishment is generally higher in regions with higher economic efficiency

In terms of the evaluation dimension system, the establishment of eco-civilization in Guangdong cities is at different levels, with most cities having a better ecological environment dimension and a poorer economic development dimension. Of the total four aspects of the assessment indicators system, the degree of eco-civilization establishment in Guangdong cities is most clearly influenced by the relative regional economic development dimension. Overall, regions with high levels of economic development scored significantly higher on the general eco-civilization building score. This is basically consistent with the results of most domestic and international literature studies that use the hierarchical analysis method to evaluate ecological civilization. Typical examples include Wang et al. (2018) [55], who evaluated the comprehensive level of ecological civilization construction and the status of the four dimensions in 21 prefecture-level cities in Guangdong Province in 2017 through the hierarchical analysis method. The results also indicate that the level of ecological civilization construction is generally higher in regions with higher economic efficiency. However, the paper was completed at the beginning of 2023, and the contextual setting was far different from that of the 18th National Congress of the Communist Party of China (CPC), and the selection of indicators was calculated by expert evaluation rather than through the “five-in-one” (economic, political, cultural, social and ecological progress) general layout highlighted in the 18th National Congress. The result is generally consistent with the direction of this paper, but the specific value of the measurement is somewhat different from that of this paper, while this paper uses GIS for the visualisation of ecological civilization level grading, which can show the research results more clearly.

### 7.2 Policy recommendations

(1) Attach great significance to the unbalanced establishment of eco-civilization in guangdong, and endeavour to improve the whole level of eco-civilization in the province

While Guangdong's overall status of eco-civilization is greater than national on average, it can be soberly seen that from the overall comprehensive score, there are still obvious imbalances in the development of cities within Guangdong, especially in cities in eastern and western Guangdong, which are far from the PRD region, and the status of eco-civilization development is clearly lower than that of other cities in the Pearl River Delta region, and the road of eco-civilization built-up in Guangdong Province is a long way to go. For this reason, we must insist on utilizing Guangdong's strengths, but also address existing inequities as a priority, in order to achieve coordinated development in Guangdong, to achieve a balanced development of eco-civilization, to take into account the natural conditions of each city according to local conditions, and to adopt appropriate policies for targeted improvement according to the social and economic basis of each city's development, for the sake of better overall eco-civilization level of the province in a timely manner.

(2) Closely monitor the changes in the state of eco-civilization in guangdong and reverse the trend of its decline

The whole of the building of eco-civilization in Guangdong Province since 2017 has shown a decreasing trend. Accordingly, it is of utmost relevance to elevate the state of our eco-civilization in Guangdong Province through the evaluation criteria of a scientific eco-civilization assessment system, which is an effective way to upgrade the level of ecological civilization in Guangdong Province. To achieve this goal, Guangdong should increase the frequency of dynamic evaluation of eco-civilization built-up, and on the basis of scientific measurement of dynamic changes, use the scores obtained by each municipality in each dimension to monitor the construction of eco-civilization under each municipality in a timely manner, so as to find out where the problems of eco-civilization achievements in each municipality lie, get to the source of the problem affecting eco-civilization establishment, co-ordinate planning, reverse the negative growth trend in a timely manner, and better realize eco-civilization.

(3) Implementing classification guidance for different cities in Guangdong Province based on the merits and demerits of eco-civilization building

From the appraisal of the eco-civilization establishment at the level of the city, each city has different strengths and weaknesses in respect of its various eco-civilization establishment dimensions, which should be used as an important basis for assortment orientation. All cities should emphasize their strengths and compensate for their weaknesses in order to improve their own eco-civilization building level, thereby boosting the level of eco-civilization built-up accordingly and gradually achieving the goal of balanced development.

(4) Focus on economic efficiency, social development dimensions, concentrate on economic development and improve income distribution policies and social security policies

In accordance with the rating results of the eco-civilization building level, except for Guangzhou and Shenzhen, which have extremely high ecological civilization ratings, all other cities in Guangdong Province are underperforming at the level of economic development or social construction. Specifically in terms of secondary indicators, the size of the gross regional product, the fairness of income distribution among residents, and the level of social infrastructure construction are the prominent weak points. The reason for this phenomenon is that at the current stage of development of the prefecture-level cities in Guangdong Province, the regionally dominated industrial structure still follows the previous crude economic development model in a large extent (Hong, 2013) [56]. Therefore, municipalities should identify the common problems that constrain their economic development and grasp the key points to solve the problems characteristic of each municipality, learn from the excellent benchmark cities, and actively take targeted measures to solve them.

(5) Strengthening cooperation among various sectors of society is one of the effective ways to achieve rapid development of eco-civilization

The achievement of eco-civilization is a systematic project that requires all relevant departments to integrate resources and coordinate development planning, publicity and education, policy measures, and institutional safeguards. In addition, coordinating the construction of hardware and software to achieve rapid growth of eco-civilization is essential (Zhang et al., 2011) [57]. The government strengthens ecological civilization legislation, incorporates ecological civilization indicators into the performance evaluation system of relevant personnel, and provides the necessary institutional guarantee for ecological civilization construction. Social media actively play the function of mass communication to strengthen the publicity of ecological civilization concepts. Scientific research institutions give full play to relevant academic research and development capabilities to strengthen the discussion, research, monitoring and advice on ecological civilization. Enterprises should invest more in the low-carbon economy, and financial institutions should increase their financial support for the development of the low-carbon economy. Each individual should consciously raise their awareness of environmental protection, starting with the small things around them. If we all work together as a society, the eco-civilization will be developed rapidly.

## 8 Limitations and future work

While the paper further explores the spatial distribution characteristics and influencing factors of ecological civilization on the basis of an objective evaluation of the level of development of eco-civilization, with a certain degree of objectivity and operability, there are still some limitations in this paper: firstly, in the selection of evaluation indicators and their influencing factors, this paper is mostly based on the availability of existing literature and data, and future research can use newer data to more systematically, dynamically and Future studies can use newer data to consider the spatial and temporal evolution of ecological civilization and its influencing factors in a more systematic, dynamic and comprehensive manner. Secondly, in terms of analysis methods, while a joint AHP and GIS can be an effective and systematic way to rate the spatial and temporal changes in eco-civilization, the AHP approach which is based on the experts' prescriptions, knowledge and judgement makes it tough to get the harmonization of scoring criteria for multiple experts, and at the same time, some qualitative indicators with a high degree of display are discarded for the sake of data availability, so the evaluation index system is not complete. Future research can incorporate subjective and objective weights in conjunction with GIS to select reasonable qualitative indicators through a more scientific methodology to rate the extent to which eco-civilization is being built more accurately and objectively. Thirdly, in terms of data classification and selection, this paper uses the spatial distribution scale of prefecture-level cities and five years of short panel data, which may affect the accuracy and robustness of the results to a certain extent. Future research can make use of continuous panel data for further in-depth study and further partitioning analysis, further subdividing the province into 7–8 types through individual and comprehensive evaluation of each dimension, and analyzing the strengths, weaknesses, opportunities and threats of each type to tailor and partition policies to local conditions (Yang et al., 2015) [11].

## Data availability statement

Data will be made available on request.

## Funding

This research is supported by the National Social Science Fund of China (21CJL007); the Humanities and Social Science Project of China’s Ministry of Education (20YJC790036); National Innovative Training Program for College Students (202311846004).

## CRediT authorship contribution statement

**Qing Guo:** Writing – review & editing, Writing – original draft, Supervision, Methodology, Funding acquisition, Conceptualization. **Mingshan Liu:** Writing – review & editing, Writing – original draft, Software, Formal analysis.

## Declaration of competing interest

The authors declare that they have no known competing financial interests or personal relationships that could have appeared to influence the work reported in this paper.
